# Prevalence of depressive symptoms and quality of life among patients with diabetes mellitus with and without HIV infection: A South African study

**DOI:** 10.4102/sajpsychiatry.v28i0.1762

**Published:** 2022-02-25

**Authors:** Yonela Qubekile, Saeeda Paruk, Farhanah Paruk

**Affiliations:** 1 Department of Internal Medicine, Faculty of Health Science, University of KwaZulu-Natal, Durban, South Africa; 2 Department of Psychiatry, Faculty of Health Science, University of KwaZulu-Natal, Durban, South Africa; 3 Department of Rheumatology, Faculty of Health Science, University of KwaZulu-Natal, Durban, South Africa

**Keywords:** HIV, type 2 diabetes mellitus, depression, quality of life, South Africa

## Abstract

**Background:**

Diabetes mellitus (DM) and human immunodeficiency virus (HIV) infection are both associated with increased risk of mood disorders and poorer quality of life (QOL). This association has not been explored in patients living with comorbid DM and HIV.

**Aim:**

To describe the prevalence of depressive symptoms and impact on the QOL in patients with DM living with and without HIV attending a public sector hospital in South Africa.

**Setting:**

A medical outpatient clinic at a state regional hospital.

**Methods:**

A cross-sectional questionnaire pilot survey was conducted amongst 101 patients with DM attending a specialist medical outpatient service. The assessment was conducted using a structured socio-demographic and clinical questionnaire, the patient health questionnaire 9 (PHQ-9) for depressive symptoms and the World Health Organization QOL scale. The HIV status was confirmed from the clinical records. The correlates of depressive symptomatology in the participants with DM living with and without HIV were identified using *t*-tests.

**Results:**

The prevalence of depressive symptoms in the participants with DM was 36%. Moderate to severe depression was associated with female gender (*p* = 0.03) and low educational level (*p* = 0.02) but not with HIV comorbidity or clinical characteristics of DM. The QOL was influenced by moderate to severe depressive symptoms (QOL in physical *p* < 0.218 and environmental *p* < 0.001 domains), but not HIV status (*p* = 0.218).

**Conclusion:**

A substantial proportion of people with DM reported depressive symptoms, which is slightly higher than the average reported in other out-patient studies. The association of depression with poor QOL highlights the need for integrated mental health access in medical outpatient services. The lack of association between comorbid HIV status and DM with depression or QOL needs to be further explored.

## Introduction

South Africa (SA) has one of the highest prevalence rates (13.7%) of human immunodeficiency virus (HIV) infections globally,^1,2^ with KwaZulu-Natal (KZN) province having the highest incidence rate in the country.^3^ Longevity amongst people living with HIV (PLWHIV) has increased in the last two decades mainly because of the availability and access to anti-retroviral treatment (ART). In combination with the changes in demographic and environmental factors, this has resulted in behavioural and nutritional transitions that have led to a higher risk of non-communicable diseases (NCDs), including diabetes mellitus (DM) and mental illness.^4^ The prevention and treatment of NCDs are still marginalised in SA because of the overwhelming prevalence of communicable diseases, such as tuberculosis and HIV or acquired immunodeficiency syndrome (AIDS).^4^

The prevalence of DM in adults (20–79 years) in Africa was 4.7% in 2019, with 19 million living with the condition. This figure is estimated to increase to 47 million by 2045.^5^ South Africa has a higher estimated prevalence of DM than the rest of Africa at 9.3%,^6^ with a recent study from KZN reporting an even higher prevalence of 14.3% in the public health sector, the majority of these patients being mainly from the urban district of eThekwini.^7^

Depression affects more than 300 million people globally (4.4%).^8^ The age adjusted rate of depression is higher in the patients with DM (8.3%) in the United States of America,^9^ whereas a Nigerian study found a much higher rate of 30%. The reasons cited for this variation included lower income levels in Nigeria and the need to provide for a higher number of dependents (children), which resulted in patients being unable to pay for their own healthcare needs.^10^ The prevalence of major depressive disorder in the South African general population is 9.8%, with the lifetime prevalence of depression in KZN being 9%.^11^ Whilst rates of depressive symptoms vary substantively in different studies,^12^ a 26% prevalence rate for depressive symptoms was reported in the cross-sectional 2014 South African Social Attitudes Survey of the general population.^13^

A higher prevalence of depression has also been observed amongst PLWHIV,^14^ with those at highest risk having not disclosed their seropositive status, lost a loved one, advanced HIV disease stage and being female.^14^ The exact prevalence of depression amongst PLWHIV remains unknown, as studies have generally not used standard diagnostic criteria according to Diagnostic and Statistical Manual (DSM) 5 or International Classification of Diseases (ICD)-10 but have relied on physician reporting or screening surveys.^15^ In contrast, a lower prevalence of depressive symptoms in PLWHIV of 20% was noted in an Ethiopian study, and the possible reason for this finding was that the participants were stable with immunological recovery.^16^ A meta-analysis from East Africa further found depression rates vary greatly and the pooled prevalence of depressive symptoms in PLWHIV was between 12.4% and 66.14% depending on the screening or diagnostic tool used.^17^ The prevalence of depressive symptoms was higher in studies using a screening tool compared to diagnostic tool.^17^ In a South African study amongst patients initiating ART, the prevalence of self-reported depressive symptoms was 25%.^18^ An association was observed between depression and duration of knowledge of HIV sero-positivity (Odds ration [OR] = 1.02, confidence interval [CI] 1.01–1.03), with those belonging to a support group being less likely to be depressed.^18^

The World Health Organization (WHO 1997) defines QoL as:

[*A*]n individual’s perception of their position in life in the context of the culture and value systems in which they live and in relation to their goals, expectations, standards and concerns. (p. 1)

It is a broad ranging and complex concept that is affected by the individual’s physical health, psychological state, personal beliefs, social relationships, and their association with the salient features of their environment.^19^

Although studies show that both HIV and DM are associated with depression and poorer QoL,^15^ this has not been explored in people living with the dual burden of both diseases. This pilot study aims to describe the prevalence of depressive symptoms in out-patients with DM living with and without HIV and explore their QoL. We also sought to describe any associations between depressive symptoms and the participants’ socio-demographic and clinical factors. We also described the QoL amongst patients with DM.

## Methodology

A descriptive, cross-sectional study was conducted from January to December 2018 in the adult medical outpatient department at King Edward VIII hospital, eThekwini District, KZN, which offers a regional level specialist service in internal medicine and psychiatry.

### Study participants

Adult patients aged 18 years and older from the medical out-patients with DM on treatment for a minimum of 12 months and living with HIV on treatment for at least 6 months were enrolled after obtaining informed consent. Control patients who were attending the same medical outpatient department, matched for gender, with DM, and who were HIV negative, as per clinical records either tested by ELISA or rapid HIV test (in the last 6 months), and consented, were also enrolled. Patients with other existing chronic medical conditions, such as hypertension and epilepsy, were excluded.

### Measures

A structured questionnaire was used to collect the socio-demographic information (age, gender, marital status, occupation, race, educational level, monthly income, and area of residence). Clinical data on the type of DM (type 1 or 2), DM complications, treatment, and control (glycated hemoglobin levels [HBA1C]) and HIV disease duration, treatment, and markers (CD4 cell count and viral load) within the last 6 months was recorded from the participants’ charts.

The Patient Health Questionnaire 9 (PHQ 9) was used which is a validated multipurpose instrument for screening, diagnosing, monitoring and measuring the severity of depression, incorporating DSM-IV diagnostic criteria, to document depressive symptoms.^20^ The PHQ 9 uses a four-point Likert scale (0 not at all, 1 = several days, 2 = more than half the days, 3 = nearly every day) to gauge responses to the questions asking about the respondents’ emotional health over the previous 2-week period. Scores on the PHQ 9 can range from 0 to 27. The scores between 0 and 4 indicate no depression, 5 and 9 mild depression, 10 and 14 moderate depression, 15 and 19 moderately severe depression, and ≥ 20 severe depression.^20^ Scores of less than 10 seldom occur in individuals with major depression, whilst that of 15 or greater usually signify the presence of major depression.^20^ For this study, 10 was used as the cut-off, as it has an overall sensitivity of 84% and a poor specificity of 72%,^20^ the tool having been used in various studies in SA.^21,22,23^

The WHO Quality of Life-BREF (WHOQOL-BREF) is a person-centred instrument for subjective assessment and based on a cross culturally sensitive concept. It consists of a number of QOL items that are concerned with the meaning of different aspects of life and how satisfactory or problematic their experience is of them. The WHOQOL-BREF is an abbreviated 26 item version of the WHOQOL-100 that contains one item from each of the 24 facets of QOL, included in the WHOQOL-100, plus two benchmark items from the general facet on the overall QOL and general health. The WHOQOL-BREF is scored in four domains that include physical health, psychological, social relations and environmental domains, and has been used in several South African studies.^24,25^

All interviews were conducted by the principal investigator (PI) after obtaining written informed consent. The PI is bilingual and conducted interviews in English and IsiZulu, with the tools being available in both.

### Sample size

The required sample size calculated was 50 patients with DM and HIV and 50 as a control with DM and no HIV. The statistical parameters used to calculate the sample size were effect size = 0.57 (medium), type I (α) error = 0.05 (the probability of falsely rejecting the null hypothesis = 5%), and type II (β) error = 0.2 (the probability of falsely retaining the null hypothesis). Statistical power = 1 – β – 1 – 0.2 = 0.8 (statistical power of 80%).

### Data analysis

Descriptive statistics were used to describe the population frequency and per cent to describe categorical variables. The frequency distributions of numeric variables were examined, and means with standard deviation or medians and inter-quartiles ranges were used as appropriate. The two groups were compared using the student *t*-test, whilst the categorical variables were compared using Pearson’s chi square test and Fisher chi square test. A significance of *p* < 0.05 was used for statistical significance testing, with the data being analysed using programme R version.

### Ethical considerations

Ethical approval for this study (BE553/17) was granted by the Biomedical Research Ethics Committee of the University of KwaZulu-Natal. Approval was obtained from the hospital and KZN provincial Department of Health. Those reporting psychological distress/requesting mental healthcare support/ with moderate to severe depression were referred to the psychiatry services at the hospital.

## Results

A total of 101 individuals participated and four refused to participate, of whom one was living with HIV and three were HIV negative. Their reasons for not participating included lack of time or other commitments. The socio-demographic and clinical characteristics of the participants with DM living with and without HIV are described in Table 1. The median age of all the participants was 54.5 (interquartile range [IQR] 48–61.3) years. The female to male ratio was 1.7:1 and most of the participants were black people (83%). People living with HIV were more likely to be younger (*p* < 0.001), black people (96%, *p* < 0.001), married (58%, *p* = 0.003), more than two-thirds lived in a township (*p* = 0.008).

**TABLE 1:** Socio-demographic and clinical characteristics of patients with diabetes mellitus with and without human immunodeficiency virus infection (*n* = 100).

Variables	Total		HIV negative		HIV positive		*p*
	*n*	%	*n*	%	*n*	%	
Age (years)†	54.5	48.8–61.3	59	51.3–65	51	47.3–55	**< 0.001**
**Gender**							0.534
Men	37	37	17	34	20	40	
Women	63	63	33	66	30	60	
**Race**							**< 0.001**
Black people	83	83	35	70	48	96	
Other	17	17	15	30	2	4	
**Marital status**							**0.003**
Single	34	34	17	34	17	34	
Married	45	45	16	32	29	58	
Divorced/Widowed	21	21	17	34	4	8	
**Residence**							**0.008**
Urban	39	39	24	48	15	30	
Rural	12	12	9	18	3	6	
Township	49	49	17	14	32	64	
**Education**							0.146
Grade 1–7	15	11	11	22	4	8	
Grade 8–12	72	72	33	66	39	78	
Tertiary	13	13	6	12	7	14	
**Occupation**							0.056
Employed	33	33	12	24	21	42	
Unemployed	67	67	38	76	29	58	
**Income**							**0.041**
< R1000	12	12	4	8	8	16	
R1001–R2500	50	50	31	62	19	38	
R2501–R5000	13	13	3	6	10	20	
R5001–R10 000	14	14	5	10	9	18	
R10 000+	11	11	7	14	4	8	
**DM**							0.538
Type 1	12	12	7	14	5	10	
Type 2	88	88	43	86	45	90	
Diabetes duration (years)†	6	3–11	6	3–11	6	3–12	0.730
DM Cardiac	15	15	9	18	6	12	0.401
Complications CVA	9	9	8	16	1	2	**0.031**
Nephropathy‡	22	22	14	28	8	16	0.161
Retinopathy	7	7	2	4	3	6	0.436
Peripheral neuropathy	7	7	0	0	7	14	**0.012**
**Treatment**							0.451
Insulin	36	36	21	42	15	30	
Oral	47	47	21	42	26	52	
Dual	17	17	8	16	9	18	
HbA1C† (mmoL/L)	7.8	6.6–99	7.8	6.6–9.4	7.7	6.6–10.5	0.461

DM, diabetes mellitus; CVA, cerebrovascular accident; HbA1C, glycated hemoglobin 1C.

† , median (IQR);

‡ , Nephropathy: Estimated glomerular filtration rate < 60 mL/min/1.73 m2 (Modification of diet in renal disease study equation).

There were no significant differences in the type of DM, DM disease duration, treatment or control, as assessed by glycated haemoglobin level between the PLWHIV and the HIV negative group. Complications related to DM were similar between the two groups, except for cerebrovascular accidents, which were higher in the HIV negative than the PLWHIV group (16% vs. 3%, *p* = 0.031). Peripheral neuropathy was only found in PLWHIV and DM (14%, *p* = 0.012).

### Prevalence of depressive symptoms

A previous psychiatric history was reported in eight (8%) participants of whom six (6%) were PLWHIV and two (2%) were HIV negative (*p* = 1.00). Sixty-four (63.3%) participants reported no to minimal depressive symptoms on PHQ 9 (0–4 score), whilst 24 (23.7%) reported mild (PHQ score 5–9) and 12 (11.8%) reported moderate to severe symptoms (PHQ score 10 or greater). There was no difference in PLHIV and HIV-negative patients in terms of severe depressive symptoms (*p* = 0.218).

The HIV comorbidity amongst people living with DM and its clinical variables, such as type and complications or control, as assessed by the HBA1C, was not significantly associated with an increased risk of moderate to severe depressive symptoms. However, female gender (*p* = 0.03) and those with lower educational level (*p* = 0.02) were more likely to have moderate to severe depressive symptoms

For the PLWHIV, the median years since HIV diagnosis was five (IQR 3–9), median CD4 cell count was 492 cells/mm³ (IQR 240–695) and 39 (78%) had a suppressed viral load. Forty-two (84%) PLWHIV were on first line ART regimen, which included Abacavir + lamuvidine + efaverens 3 (6%), Zidovudine + lamuvidine + efaverens one (2%), Tenofovir + emtricitabine + efaverens 38 (76%), and eight (16%) were on second line/other ART regimens. As only four PLWHIV who were all virologically suppressed and on three different regiments had a PHQ 9 score of greater than 10, we did not explore variables such as CD4 cell count, viral load, antiretroviral treatment and treatment duration as sub-groups made analysis difficult because of the limited sample sizes.

Table 2 summarises the data comparing the participants with DM with no or mild depressive symptoms to those with moderate to severe depressive symptoms and their associations with socio-demographic and clinical factors, including comorbid HIV status, which was not statistically significant.

**TABLE 2:** Association of depressive symptom score with socio-demographic and clinical variables of diabetes mellitus and human immunodeficiency virus (*n* = 100).

PHQ-9 total score	Total		PHQ ≤ 9.9		PHQ ≥ 10		*p*
	*n*	%	*n*	%	*n*	%	
Age (mean ± SD) years	53.7	11.1	53.3	11.2	56.6	10.6	0.343
**Gender**							**0.030**
Male	37	37	36	40.9	1	8.3	
Female	63	63	52	59.1	11	91.7	
**Marital status**							0.172
Single	34	34	32	36.4	2	16.7	
Married	45	45	40	45.5	5	41.7	
Divorced/widowed	21	21	16	18.2	5	41.7	
**Occupation**							0.327
Employed	33	33	31	35.2	2	16.7	
Unemployed	67	67	57	64.8	10	83.3	
**Race**							0.424
Black people	83	83	74	84.1	9	75	
Other	17	17	14	15.9	3	25	
**Education**							**0.020**
Grade 1–7	15	15	10	11.4	5	41.7	
Level Grade 8–12	72	72	65	73.9	7	58. 3	
Tertiary education	13	13	13	14	0	0	
**Income**							0.406
Under R1000	12	12	10	11.4	2	16.7	
R1001–R2500	50	50	44	50	6	50	
R2501–R5000	13	13	10	11.4	3	25	
R5001–R10 000	14	14	14	15.9	0	0	
R10 000+	11	11	10	11.4	1	8.3	
**DM**							0.351
Type 1	12	12	12	13.6	0	0	
Type 2	88	88	76	86	12	100	
**Treatment**							0.503
Insulin	36	36	30	34	6	50	
Oral	47	47	43	49	4	33	
Dual	17	17	15	17	2	17	
Duration of DM (years)†	6	3–11	6	3–11	10	3–11	0.608
HbA1c (mmoL/L)†	7.8	6.6–10	7.7	6.6–9.8	8.6	7.6–11.2	0.168
DM Cardiac	15	15	13	15	2	17	1.000
Complications CVA	9	9	7	8	2	17	0.294
Nephropathy	22	22	19	22	3	25	0.713
Retinopathy	7	7	6	7	1	8	1.000
Peripheral neuropathy	7	7	7	8	0	0	0.594
**HIV status**							0.218
Negative	50	50	42	48	8	67	
Positive	50	50	46	52	4	33	

DM, diabetes mellitus; CVA, cerebrovascular accident; PHQ, Patient Health Questionnaire; SD, standard deviation; HbA1C, glycated hemoglobin 1C.

† , median (IQR).

### Quality of life and association with depressive symptoms

The WHO QoL BREF score for each domain did not differ significantly between the PLWHIV and HIV-negative participants, as noted in Table 3. However, those who had moderate to severe depressive symptoms had a poorer QOL in the physical and environmental domains compared to those with no or mild depressive symptoms, as summarised in Table 4.

**TABLE 3:** Association of quality of life in patients with diabetes mellitus with and without human immunodeficiency virus infection.

QOL sub-domains	Total		DM & HIV negative		DM & HIV positive		*p*
	*n*	±SD	*n*	±SD	*n*	±SD	
Total physical †	23.6	5.6	23	6	24.1	5.2	0.592
Total psychological †	20.2	2.4	20.6	2.6	19.9	2.2	0.592
Total environmental †	27	4.9	26.9	5.7	27.6	3.9	0.766
Total social †	7.5	1.5	7.6	1.6	7.42	1.4	0.161

† , mean (±SD).

**TABLE 4:** Association between depressive symptom score (patient health questionnaire 9) and quality of life in total cohort of patients with diabetes mellitus with and without HIV infection.

QOL domains score	Total		PHQ ≤ 9		PHQ ≥ 10		*p*
	*n*	±SD	*n*	±SD	*n*	±SD	
Total physical†	23.6	5.6	24.5	4.9	16.4	5.8	**< 0.001**
Total psychological†	20.2	2.4	20.5	2.2	18.1	2.9	0.005
Total social†	7.5	1.5	7.7	1.4	6.6	2	0.092
Total environmental†	27.3	4.9	28	4.4	22.1	5.3	**< 0.001**

† , mean (±SD).

## Discussion

This is a pilot case control study from SA to determine the prevalence of depressive symptoms in patients with DM living with and without HIV and the impact of depressive symptoms on the QOL. The study’s key findings are that at least one-third (36%) of the participants with DM reported depressive symptoms, which were mainly undetected. Whilst comorbid HIV infection in DM (type, complications or control) was not associated with moderate to severe depressive score, moderate to severe depressive symptoms were associated with gender, educational level and poorer QOL (physical and environmental domains) amongst all people with DM.

The prevalence of depression in our study was higher than that reported in the South African general population at 9.7% for lifetime and 4.9% for the 12 months prior to interview.^26^ A recent study similarly showed a much higher prevalence of depression (46%) amongst patients with type 2 DM attending a specialist diabetic clinic, which again suggests vulnerability to depressive symptoms amongst this group.^23^ Reasons for the variation in the prevalence of depression may include use of different measurement tools and cut-off points.^27^

The current study finding, that depressive symptoms were associated with female gender and lower educational level, is supported by the literature.^8,23,28^ Tomlinson and colleagues showed that the prevalence of depression was much higher amongst females, who were 1.75 times more likely to experience lifetime depression than males, and was significantly higher amongst those with low level of education.^26^ The Hunt study examined if a higher educational level protects against anxiety and/or depression and if this protection accumulates with time. The results showed that low educational levels were significantly associated with depression, whilst higher educational levels had a protective effect that accumulates throughout life.^29^

The findings of this pilot study failed to establish any association between depressive symptoms and clinical features of HIV or DM, which suggest that they may not be the most pressing risk factors in individuals receiving chronic specialist medical care, and that socio-demographic factors, such as gender and educational level, remain driving factors that influence mood disorders. There is well-documented evidence to support the interaction and outcomes between DM and depression, which is mediated by social contexts, especially amongst low-income countries.^30^ Whilst Tesfaw and colleagues found that patients with stage III HIV were more likely to be depressed, they also identified that psycho-social factors, such as HIV stigma, poor social support and poor medicine adherence, increased the risk of depression.^31^ A Nigerian study found that 56% of PLWHIV had depressive symptoms, which these were also associated with psycho-social factors, such as female gender, below average schooling and poor economic status.^28^ This suggests the need to consider providing additional psychosocial support and mental health care access for the most vulnerable in our communities.

The association of QoL with the PHQ-9 score showed statistical differences in the physical and environmental domains, which suggests that comorbidity of HIV and DM was not impacting on QoL, but that mental health problems were. These findings are consistent with a previous study, which showed that all four domains of QoL were associated with depression in patients with Type 2 DM.^32^ Deshmuck and colleagues also noted that in PLWHIV, depression was associated with significantly lower QoL, particularly in the social and environmental domains.^33^ An Indonesian study reported a strong correlation of higher depression scores associated with lower QoL in PLWHIV.^34^ Finally, a study conducted in Uganda reported that although QoL improved over time for PLWHIV, it was associated with depression, low education level and female gender.^35^ Thus, it is essential to screen and treat underlying mood disorders in patients with a medical disorder, as it could be impacting on the QoL. The use of a simple screening tool, such as the PHQ 9, could be an initial important step towards addressing issues related to QOL.

### Limitations

The study has some limitations because of the fact that it took place at a single clinical site with a small sample size, which limits its generalisability, although it provides a snapshot of the current clinical public sector characteristics of patients with DM living with and without HIV. The study is urban- and hospital-based, so the findings may not be generalisable to other settings, and relies on self-reporting of symptoms, with the potential for patients under/over reporting symptoms. In addition, the poor specificity and sensitivity of PHQ 9 for depression is noted when using a cut-off score of 10 on this screening tool. There is a need for a longitudinal larger community-based study to further explore the factors associated with depressive symptoms in this population.

## Conclusion

The high prevalence of undetected depressive symptoms associated with gender and educational level, and that those with depression reported poorer QOL in two domains amongst patients with DM with and without HIV, highlight the importance of diagnosing and treating for underlying mood disorders in patients with chronic medical illnesses. This indicates the need for efficient mental healthcare interventions to be integrated into routine clinical care in medical outpatient services to treat the person, and not just their medical condition.
